# Improved PET/MRI accuracy by use of static transmission source in empirically derived hardware attenuation correction

**DOI:** 10.1186/s40658-021-00368-5

**Published:** 2021-03-08

**Authors:** Adam Farag, R. Terry Thompson, Jonathan D. Thiessen, Frank S. Prato, Jean Théberge

**Affiliations:** 1 Lawson Health Research Institute, Imaging Division, London, Ontario Canada; 2 Department of Medical Biophysics, Western University, London, Ontario Canada; 3 Department of Medical Imaging, Western University, London, Ontario Canada; 4 St. Joseph`s Health Care, Diagnostic Imaging, London, Ontario Canada; 5 Lawson Imaging Division, St. Joseph’s Health Care London, 268 Grosvenor St., PO Box 5777, STN B, London, ON N6A 4V2 Canada

**Keywords:** PET/MRI, Cardiac imaging, Hardware attenuation map, Transmission-based attenuation correction

## Abstract

**Background:**

Accurate quantification of radioactivity, measured by an integrated positron emission tomography (PET) and magnetic resonance imaging (MRI) system, is still a challenge. One aspect of such a challenge is to correct for the hardware attenuation, such as the patient table and radio frequency (RF) resonators. For PET/MRI systems, computed tomography (CT) is commonly used to produce hardware attenuation correction (AC) maps, by converting Hounsfield units (HU) to a linear attenuation coefficients (LAC) map at the PET energy level 511 keV, using a bilinear model. The model does not address beam hardening, nor higher density materials, which can lead to inaccurate corrections.

**Purpose:**

In this study, we introduce a transmission-based (TX-based) AC technique with a static Germanium-68 (Ge-68) transmission source to generate hardware AC maps using the PET/MRI system itself, without the need for PET or medical CT scanners. The AC TX-based maps were generated for a homogeneous cylinder, made of acrylic as a validator. The technique thereafter was applied to the patient table and posterior part of an RF-phased array used in cardiovascular PET/MRI imaging. The proposed TX-based, and the CT-based, hardware maps were used in reconstructing PET images of one cardiac patient, and the results were analysed and compared.

**Results:**

The LAC derived by the TX-based method for the acrylic cylinder is estimated to be 0.10851 ± 0.00380 cm^−1^ compared to the 0.10698 ± 0.00321 cm^−1^ theoretical value reported in the literature. The PET photon counts were reduced by 8.7 ± 1.1% with the patient table, at the region used in cardiac scans, while the CT-based map, used for correction, over-estimated counts by 4.3 ± 1.3%. Reconstructed in vivo images using TX-based AC hardware maps have shown 4.1 ± 0.9% mean difference compared to those reconstructed images using CT-based AC.

**Conclusions:**

The LAC of the acrylic cylinder measurements using the TX-based technique was in agreement with those in the literature confirming the validity of the technique. The over-estimation of photon counts caused by the CT-based model used for the patient table was improved by the TX-based technique. Therefore, TX-based AC of hardware using the PET/MRI system itself is possible and can produce more accurate images when compared to the CT-based hardware AC in cardiac PET images.

## Introduction

Hybrid positron emission tomography (PET) and magnetic resonance imaging (MRI) systems are becoming increasingly important in cardiovascular diagnostic imaging [27]. In cardiac PET/MRI, accurate measurement of PET activity is necessary for evaluating cardiac function [28] and inflammation [37, 38], and grading tumors based on their standardized uptake values (SUV) [24]. One aspect that affects the accuracy of PET quantification is the attenuation of photons absorbed by fixed or mobile hardware present during scans, such as radio frequency (RF) arrays, the patient table, and headphones. The adverse effects of such hardware on PET quantifications has been reported numerously in publications; for example, the patient table of the PET/MRI system causes up to 18.7% loss of PET true-counts [14], while RF resonators, for the same system, caused true-count losses varying between 3 and 14% [2, 11, 12, 19, 25, 31, 35].

In addition to the patient attenuation correction (AC) map, it is necessary to include AC maps of hardware present in the PET field of view (FOV) during the scan. However, unlike patient AC maps, which can be determined using an MRI attenuation correction acquisition (MRAC) [22], hardware is often invisible to MRI. To overcome such hardware attenuation, it is often required to prospectively redesign some hardware (i.e. rigidly fixed RF arrays) for simultaneous PET/MRI, aiming to reduce their photon attenuation [12]. However, other hardware, such as the patient table, would be challenging to redesign, and hence, techniques to correct their attenuation are still a topic of study.

In a PET-only system, the challenge of hardware AC is addressed through transmission scan that can be carried out after patient injection with the emission tracers or, simultaneously, using multiple transmission sources in transmission-emission scans fashion [3]. In this setting, a rotating rod containing a radioactive source either emitting positrons (Ge-68/Ga-68) [7] or a source emitting a single photon (Cesium-137) [10] is utilized to map the hardware for attenuation. Meanwhile, in an integrated PET/CT system, this challenge is simpler, since hardware is visible on the medical computed tomography (CT) scan, and hence, CT images can be used to map hardware attenuation. To produce CT-based AC of hardware, the CT-HU are converted and scaled into linear attenuation coefficients (LAC) of PET annihilation photon energies (511 keV), which are estimated by either a bilinear transformation model from a single medical CT energy (140keV) [5], from a dual-energy CT to PET energy level [6, 16, 21, 30], or a multi-energy medical CT imaging [15]. The bilinear model is based on different slopes for air-water (HU < 0) and water-bone (HU > 0). Although the bilinear transformation model of CT-HU to PET LAC is optimized for densities ranging from air-to-bone, it is still employed in AC of hardware on PET/MRI system. This led to an attempt to improve the model and address material with high atomic number (Z) [29]; nevertheless, the group reported general limitations when larger amounts of highly attenuating materials are to be corrected. While CT-based attenuation correction is the silver-standard for hardware AC of PET images, with CT-based AC, images still have errors up to 4.7% [34]. Causes of these errors invoke the use of a broad polychromatic spectrum X-ray at lower photon energies for CT image acquisition, unlike PET image acquisition, which is monochromatic. Hence, the transformation of HU to LAC at 511 keV requires extensive energy mapping. Additionally, the polychromatic X-ray in CT can cause beam-hardening artefacts, specially with materials with a high atomic number (e.g. electronics found in hardware), which further degrades the measurement of accurate attenuation coefficients [15].

With this in mind, transmission (TX)-based AC has an advantage as an alternative to CT-based AC methods for PET/MRI systems. The transmission-based AC is based on acquiring a “blank” scan (without the hardware), and a transmission scan (with the hardware), where the ratio of the two scans, in simple terms, is the LAC. The method based on a rotating radioactive source “rod” to produce AC maps on PET and PET/CT systems has been used for decades [21, 39]. However, it is not possible to incorporate the rotating rod into the PET/MRI system, due to the complexity of the system design. Furthermore, the ability to reduce radioactive dose with MRI in PET/MRI would be diminished if a radioactive source is used to measure the tissue and hardware AC. It would also increase the scan time, which would further diminish the advantages of simultaneous PET/MRI. As an alternative to the rotating rod, liquid fluoro-deoxy-glucose (^18^F-FDG) was injected in an annulus-shaped phantom tube that circumfused the hardware and was used as a transmission source [4, 23, 26, 33]. However, this alternative transmission method required more complex algorithms to allow the simultaneous reconstruction of emission and transmission data.

This study aims to propose TX-based AC using a “uniform and static” radioactive source (Ge-68 rod) for generating AC maps for hardware used in PET/MRI systems as an alternative to using medical CT or rotating radioactive source techniques. Thus, the novelty of this study is to develop a new technique using a uniform-static source for LAC estimation of hardware like a patient table or RF arrays.

## Materials and methods

### Theory

The linear attenuation coefficient (*μ*) describes the fraction of photons from a monoenergetic gamma ray that is attenuated by material, per unit path length (*l*). This is expressed as the ratio of the number of photons detected (*I*) by the PET detector along the line of response (LOR) with the hardware in place, to the number of photons detected (*I*_*0*_) from a blank scan (without the hardware), and is described as:

1 $$ \frac{I}{I_0}={e}^{-\mu l}\left[1\right] $$

In the case of a PET TX scan, for coincident detection of photon at any detector, Eq. (1) can be written in a discreet form of Lambert’s law:

2 $$ \frac{I(p)}{I_0(p)}={e}^{-\sum \limits_p{\mu}_{i,j}(p).{l}_{i,j}(p)}\left[2\right] $$

where *i* is the detector index, *j* is the voxel index, and *p* is the hardware voxel position in the PET-FOV. Since both LAC and path length are functions of position, in a PET-only scanner, a rotating rod at multiple locations can provide the position of each point of the hardware, and the partial path length is known. In this study, we chose to use a known position of the radioactive source (Ge-68 rod), at the centre of the bore (along the *x*, *y* and *z* axes), this together with knowing the locations of the detectors [1] and 3D data profile of the hardware, the partial path length at each point in the hardware can be calculated. Therefore, the hardware profile was registered to known voxel size and voxel positions, in which, together with the known position of the radioactive source, each position indices have a partial path length at a given LOR which can be estimated using the following relation:

3 $$ {l}_{i,j}(p)=\sum \limits_p{d}_i(p).{b}_j(p)\left[3\right] $$

where *d* is the voxel size and *b* = 1 when a voxel area is integrated into the LOR and contains a hardware profile, otherwise is equal to 0. Assuming that the hardware is homogeneous enough, density-wise, the formulation is applied for only one unknown. Since position indices and partial path length projected are known, LAC can now be estimated and assigned to each voxel.

### Acrylic cylinder and TX-fixture

**Fig. 1:**
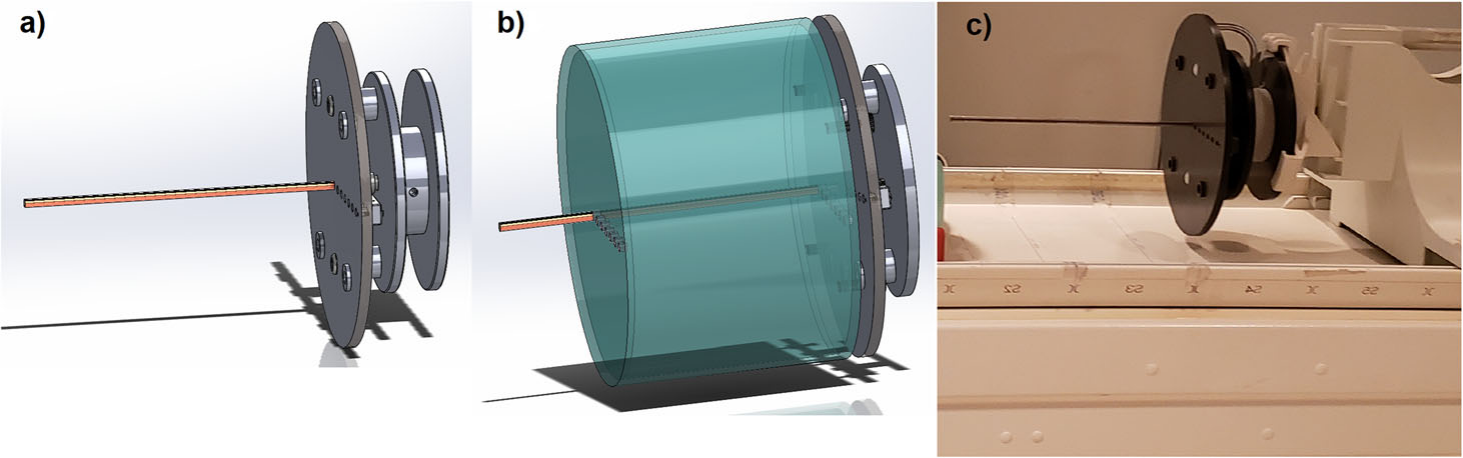
The apparatus (TX-fixture) and setting used in PET acquisitions, **a** schematic of the TX-fixture showing the radioactive Ge-68 rod positioned in the centre, during blank scan, **b** both the acrylic cylinder phantom and the rod attached to the TX-fixture, and **c** the QC-daily phantom holder securing the TX-fixture to achieve a reproducible position on the patient table

In order to test the proposed method accuracy, one must confirm the LAC value measured by the method with a known theoretical LAC value of a known material. For this purpose, a phantom and a fixture to hold both the phantom (empty acrylic cylinder) and the radioactive source were reconstructed. To align the source in a known position and maintain consistent position in all scans, a fixture, namely a TX-fixture, was developed to hold the rod with better than 1-mm tolerance. The TX-fixture utilizes the vendor’s daily-quality control (QC) phantom holder for mounting to the table at any position. The TX-fixture was designed to hold and secure multiple or single rod(s) of Ge-68 (37 MBq; Sanders Medical, USA), where the rod has an outer diameter of 8 mm and a length of 280 mm. It also permits attaching the cylinder and the rod together or individually as seen in Fig. 1, a, b, and c. Cylinder material selection criteria as an attenuation validator included the following: known LAC value at 511 keV, geometric symmetry, and synthetic/controllable chemical contents. We chose acrylic with purity of 99.99% and known chemical composition (C_5_O_2_H_8_). The cylinder was hollow with the exception of a series of circular holes at its bases where the rod source was positioned. The cylinder was manufactured with 9-mm wall thickness, 210-mm length, and 310-mm diameter (Fig. 1b).

### TX-based AC

**Fig. 2:**
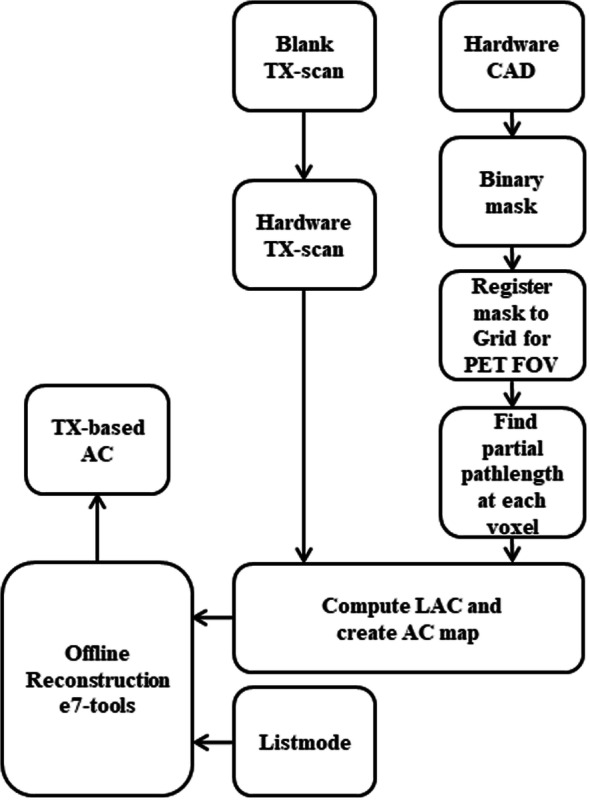
Flow chart describing the process of generating hardware TX-based AC map using the PET/MRI scanner and a static radioactive source

In Fig. 2, a flow chart of the steps followed to produce TX-based AC map is shown. A set of PET TX acquisitions was performed post QC-daily normalization routine on a 3.0T PET/MRI system (Biograph mMR Software Version VE11P, Siemens Healthineers, Erlangen, Germany). The TX acquisition included (1) a blank acquisition (cold) of the rod, centred by the TX-fixture in the bore, with no-table or other hardware in the PET-FOV, and therefore, photon counts can be used as a reference and becomes the target to reach; (2) a TX scan (cold) with the cylinder attached to the TX-fixture with the rod in the same position as the blank TX scan. The time between the two scans, blank and with hardware, was approximately 7 min resulting to ~ 0.0009% decay of the Ge068 activities, and therefore, decay correction was ignored. For patient MRAC, a two-point Dixon, 3D spoiled gradient sequence using TR = 4.14 ms, TE = 2.51 ms and 1.2 ms, and matrix size = 240 × 126 × 127, was acquired to generate a tissue AC map with segmentation of air, lung, water, and fat. A PET acquisition was performed for 5 min with matrix size 344 × 344 × 127 at a resolution of 2.09 × 2.09 × 2.02 mm^3^. PET reconstruction on the scanner was performed by an Ordinary Poisson Ordered Subsets Expectation Maximization (OP-OSEM) algorithm [9] including 3 iterations, 21 subsets, and a 4-mm Gaussian filter. For derivation of the TX-based LAC of the cylinder, a 3D-CAD profile was scaled to the same parameter as that of the PET acquisition and registered to image voxel. The partial path length of each voxel along the LOR to each detector was calculated from a simulated PET-FOV of the scanner geometry (8 rings each including 56 detector blocks at a rotating angle of ~ = 6.4°) [1]. Positioning the rod at the centre of both the PET-FOV and the cylinder allowed for symmetrical and homogeneous conditions, and therefore, the distance between the source and a detector could be treated as a constant at a given plane number. Therefore, partial path lengths were assumed to be unchanged across planes for a given voxel. The resultant TX-based AC map was used with list-mode data, from the TX scan of the cylinder, to reconstruct the PET images.

Scatter correction estimation was not in the scope of the formulation in this manuscript. Errors on scatter correction of the hardware were assumed to have a non-dominant effect [17] on the results, compared to tissue scatter correction estimates. All generated AC images were reconstructed using the vendors’ e7-tools, which corrected for scattering based on single scatter simulation (SSS) [36].

All offline PET reconstructions using TX-based AC in this study were performed using the manufacturer’s offline reconstruction tool (e7-tools, Siemens Molecular Imaging, Knoxville, USA).

### Theoretical LAC of acrylic

To verify the accuracy and validity of the TX-based LAC derived for the cylinder, we used the “un-renormalized” Scofield (1973) theoretical values of the photo-effect cross section for all elements with atomic number *Z* ≥ 2 of the acrylic chemical composition. The analytical results used for *Z* = 1 are the same as those used in the [18]) compilation. LAC of acrylic was mapped at energy levels in the range between 0.1 and 1.0 MeV, where LAC was subsequently interpolated at 511 keV using a fourth-order polynomial fitting.

### CT-based AC

The cylinder was scanned using a medical Dual-Energy CT (GE Healthcare, Discovery CT750 HD, Waukesha, USA), with the following parameters: tube voltage = 140 and 70, tube current = 630 mA, pitch = 0.515625 degree, FOV= 60 × 60 cm^2^, and exposure time = 912 s. The HU were converted to LAC at 511 keV using a bilinear model [6]. The CT-based AC map was used together with the nonattenuation-corrected (NAC) of the cylinder to reconstruct the cylinder AC images offline.

### Validation of the technique

The validation criteria of the technique were identified as the agreements of PET total photon counts and distribution between both TX-based AC of the cylinder and the blank AC. We have selected an agreement threshold of ≤ 1% globally, aiming to improve attenuation accuracy over the CT-based AC method which is currently achieving (2.7%). After validating the technique, it was applied to the rigid/fixed posterior part of the 32-channel cardiac array described in [13] and the patient table.

### Posterior array

The same procedure as described above was performed to derive the hardware AC map of the rigid/fixed posterior part of the cardiac array. However, the hardware profile was derived from a 3D-CT map [12]. The posterior profile was created by transforming the maps into binary values where each voxel containing posterior information was assigned 1, otherwise 0. In this TX scan, the posterior array was placed in the bore and positioned on the patient table rails. The targeted area, including the elements, were centred in the PET-FOV, and with this setting, the TX scan of the posterior array, without the patient table, matched the location of the posterior array when used for cardiovascular imaging.

### Patient table

We chose the patient table area in this study to be centred between the S2 and S3 markings on the patient table which normally relates to the position of the elements of the spine matrix for this scanner. This allowed a consistent and match of the locations of the posterior array, table, and cardiac patient during AC reconstruction using the TX-based AC maps. For this setting, the daily-QC phantom holder was mounted to the patient table, with no other object present, and the TX scan was performed as described earlier.

### In vivo reconstruction

A patient was recruited, with written informed consent according to a research ethics protocol, approved by the Western University Health Science Research Ethics Board (HSREB) (protocol ID R-20-069). The volunteer was injected with 462MBq of ^18^F-FDG tracer for a PET/CT scan first, and then, the PET/MRI scan was started 3 h and 15 min later. However, none of the PET/CT data was used in this study. Simultaneously with a two-point Dixon acquisition, as described earlier, a 3D PET acquisition was performed using the same parameters as those used for the TX scans. A set of cardiovascular MRI scans were acquired, but we report here only on two acquisitions related to this study and analysis. A 2D axial half-Fourier single-shot turbo spin-echo (HASTE) MRI was acquired using imaging parameters TR/TE = 1000 ms/82 ms, 20 slices, slice thickness (ST) = 6 mm, matrix = 256 × 154, FOV = 380 × 380 mm, and flip angle (FA) = 139°.

Positions of TX-based maps for subject, table, and array were matched. This is done by acquiring the in vivo image at the centre of a known location, between S2 and S3, same as the table and array regions used in calculating the TX-based LAC. In vivo PET list-mode data was reconstructed offline with the TX-based AC maps for both posterior array and patient table, using the e7-tools software. The AC images produced offline were analysed and compared to the vendor-provided, CT-based AC images produced by the PET/MRI system itself.

### Data analysis

In all analysis, hardware NAC data was compared to blank NAC data and hardware AC data was compared to blank AC data, while for in vivo imaging, TX-based AC was compared to CT-based AC. Hardware images reconstructed from the TX-based AC map were quantitatively compared to its corresponding CT-based AC images and blank images using relative percentage difference (RPD) defined voxel-wise as (Blankdata – CT-based_data_ / Blankdata) * 100% and (Blank_data_ – TX-based_data_ / Blank_data_) * 100%. Furthermore, distribution of photon counts was examined using histogram analysis. In order to examine the effect of both TX-based AC and CT-based AC on cardiovascular quantification, 17-segment polar plots of short-axis PET images were produced according to the American Heart Association (AHA) standard for cardiac polar plots [8]. The segment values, which cover from base to apex of the heart, were compared for both AC techniques.

All computation of this work was performed using Matlab 9.7.0.13 (The MathWorks, Natick, MA, USA).

## Results

### TX-based AC map and validation of LAC

The TX-based procedure described in this work produced an AC map of the cylinder material with an average LAC of 0.10851 ± 0.00380 cm^−1^. The theoretical Z-based LAC derived from the chemical composition of acrylic described by [18]) was estimated with and without coherent scattering and found to be 0.11052 ± 0.00311 cm^−1^ and 0.10698 ± 0.00321 cm^−1^, respectively. The coefficient of determination of the fitted fourth-order polynomial function is calculated to be *R*^2^ = 0.9995. The percentage difference between the theoretically derived LAC and measured LAC, using TX-based technique, was found to be between 1.7 ± 0.3% and 1.3 ± 0.3% which was not statistically significantly different (*p* = 0.7060).

### TX-based AC and CT-based AC for hardware

**Fig. 3:**
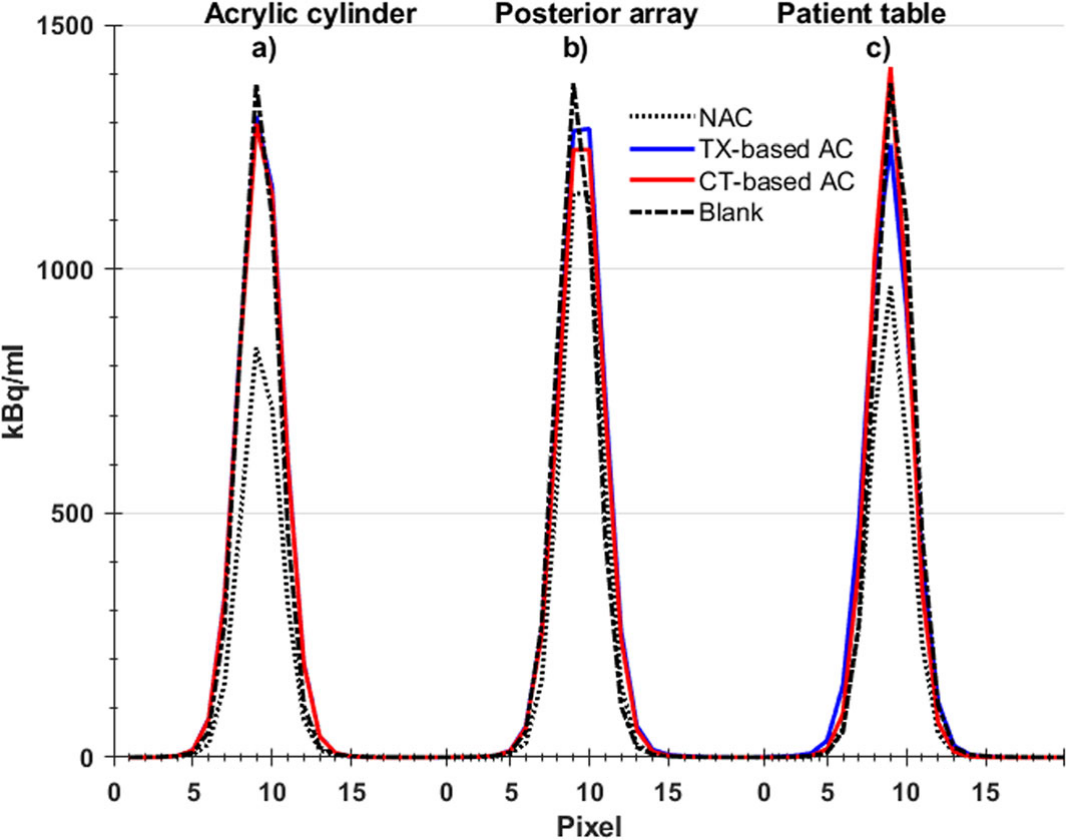
Line plot of a profile from pixels centred on the PET image produced. For the three-hardware configurations examined in this study, left line plot results from acrylic cylinder with NAC PET, TX-based AC PET, CT-based AC PET, and blank (black-dashed line). The same order of plots is repeated for the posterior array and patient table, respectively

Figure 3 shows the line profiles, from the centre of the PET image resulted from scanning the Ge-68 rod with and without different hardware in the PET-FOV. The line profiles are arranged in three clusters; each is related to the hardware under examination and includes a line profile for NAC PET and two-line profiles for AC PET reconstructed with TX-based and CT-based methods. The cylinder scans produced the line profile seen in Fig. 3 a and represent NAC PET, the TX-based AC PET reconstructed offline and CT-based AC PET (produced by the CT scanner in a separate CT scan of the cylinder). The RPD values comparing the two AC PET reconstructions are reported in Table 1, where TX-based AC PET mean and standard deviation (SD) was different from blank by 0.4 ± 1.4 %, while the RPD of mean values in the case of CT-based AC was − 1.9 ± 0.8 %.

**Table 1:** Relative percentage difference for non-corrected and corrected images of hardware to nonattenuation-corrected and attenuation-corrected image of blank scan. In vivo data from TX-based AC and CT

	NAC	TX-based AC	CT-based AC	TX-to-CT
	Mean ± SD	Mean ± SD	Mean ± SD	Mean ± SD
**Acrylic cylinder**	2.2 ± 1.1	0.4 ± 1.4	− 1.9 ± 0.8	**–**
**Posterior array**	1.6 ± 0.6	0.5 ± 1.5	0.7 ± 0.5	**–**
**Patient table (S2–S3)**	8.7 ± 1.2	− 0.7 ± 1.4	− 4.3 ± 1.3	**–**
**In vivo**	**–**	**–**	**–**	4.1 ± 0.9

Figure 3b shows the NAC and both AC PET line profiles from the posterior array results using the TX- and the CT-based AC correction. As reported in Table 1, the TX-based AC map, for posterior array, produced PET images with RPD of 0.5 ± 1.5%, while CT-based AC produced RPD of 0.7 ± 0.5%. The posterior array was estimated to attenuate about 1.6 ± 0.6% comparing between NAC of the posterior array and the NAC of blank. In Fig. 3c, the results from the patient table are showing the NAC data compared to both AC techniques. Table 1 reported the RPD value between the NAC PET of the patient table and the NAC PET of the blank, which was found to be 8.7 ± 1.2%, which is consistent with what has been reported earlier [14]. The patient table TX-based AC reported to produce global mean RPD of − 0.7 ± 1.4% while for the CT-based AC the RPD was − 4.3 ± 1.3%.

**Fig. 4:**
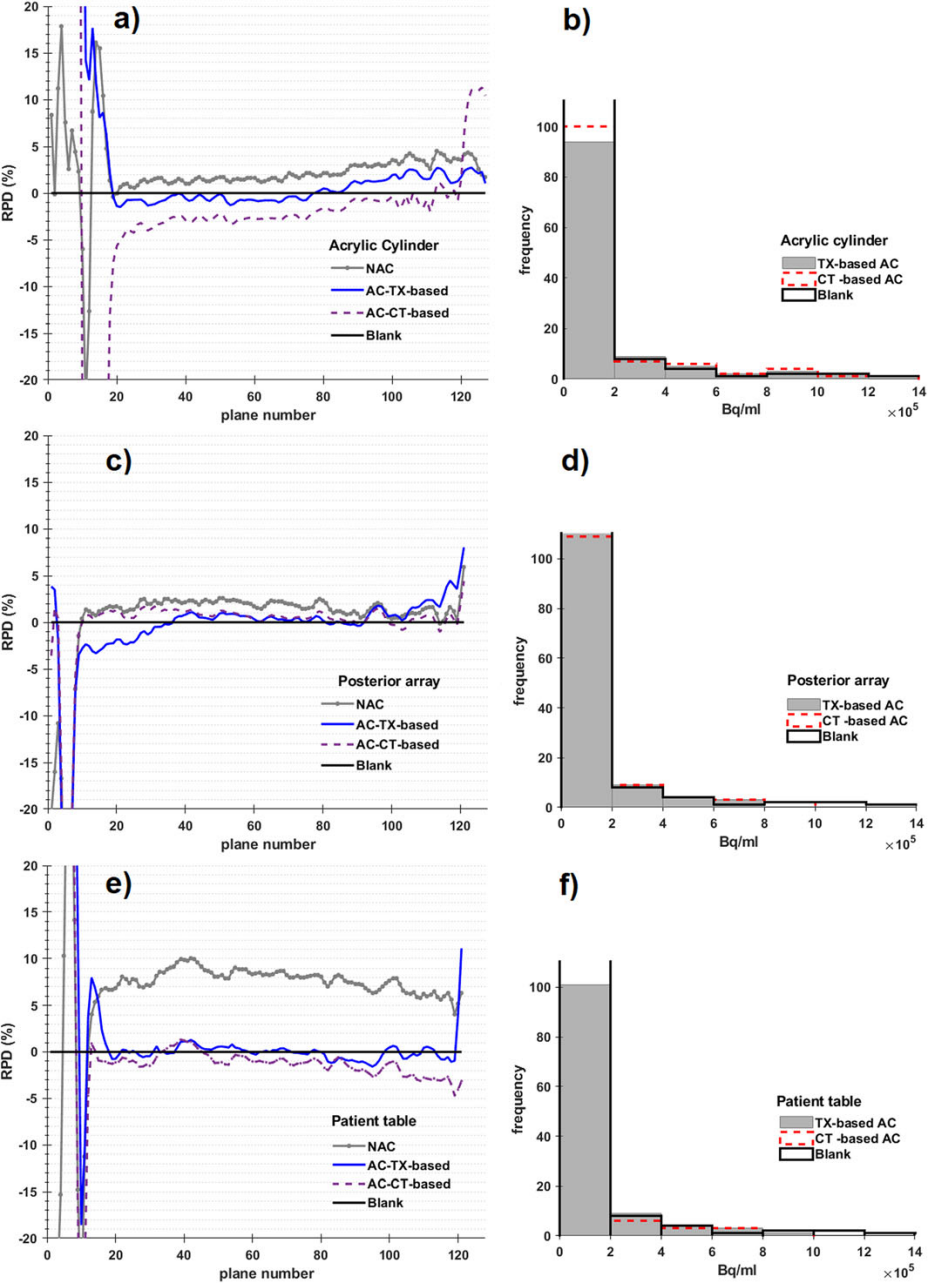
RPD of NAC, TX-based AC, and CT-based AC PET images for the acrylic cylinder, posterior array, and patient table are shown in **a**, **c** and **e,** respectively, compared to blank. Distribution of photon counts are displayed in histograms in **b**, **d**, and **f** comparing TX-based AC to CT-based AC and blank

In general, Fig. 4 shows both quantitative and qualitative analysis of both TX-based and CT-based AC PET data and comparison to blank data. In Fig. 4 (a, c, and e), the RPD between blank images and its corresponding images of each AC technique or NAC is displayed for each transaxial plane number. In Fig. 4 (b, d, and f), the distribution of photon counts per second and their frequency for each hardware, acrylic cylinder, posterior array, and patient table, respectively, are shown by histogram plot. Notably, the CT-based AC PET distribution displays more frequent counts per second at lower values, over TX-based AC PET. Likewise, the distribution of TX-based AC PET is closer to those produced from blank in the case of the patient table, although this might not be apparent (Fig. 4f), as the dashed red line belonging to CT-based plot are only covered by the black lines of the blank data. The number of voxels with lower PET concentrations increased from the acrylic cylinder to the patient table, while voxels with maximum concentrations are decreased from the patient table to the cylinder. The increase of the number of voxels with lower concentration could be indicative of more background noise produced from the hardware’s presence, and the more complicated the geometry, the more background noise. The number of voxels with lower concentrations in the TX-based AC images was closer to the blank values and is lower for all three hardware. The percentage mean difference between AC and NAC blank were estimated to be in the range of 11 to 18%.

### In vivo data

**Fig. 5:**
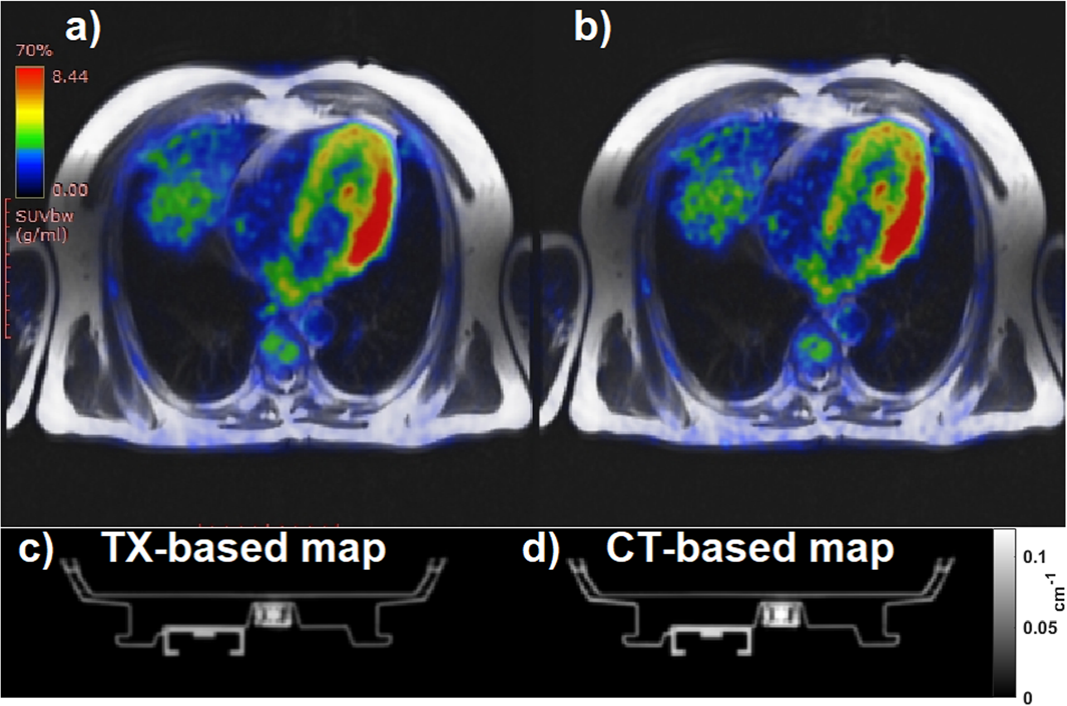
Fused images (**a** and **b** top) of the 2D-HASTE MRI to 18F-FDG PET for a volunteer. **a** The offline reconstructed PET with the scanner using hardware TX-based maps (both, patient table and posterior part of the array). **b** The same PET data, reconstructed using hardware CT-based maps. The lower row of the figure (**c** and **d**) shows, respectively, the TX-based and the CT-based maps of the patient table

**Fig. 6:**
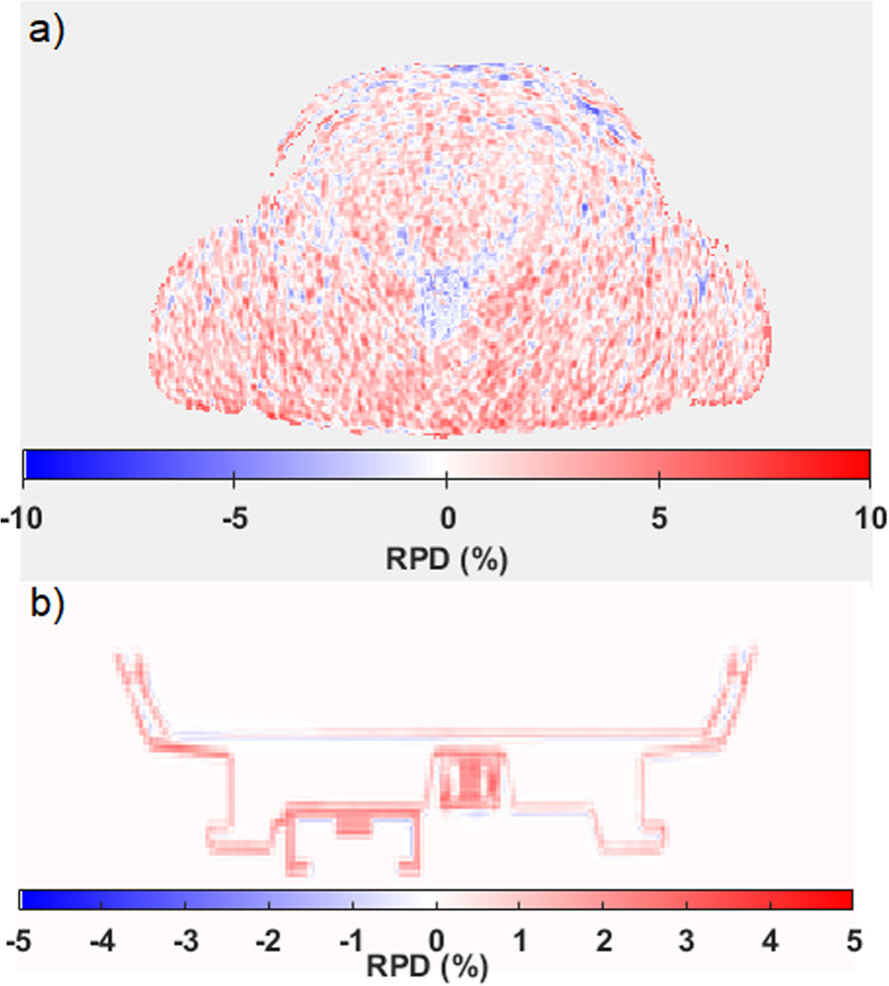
**a** RPD map of PET data resulted from comparing TX-based AC to CT-based AC shown in Fig. 5 (**a**, **b**); **b** the RPD map of the hardware AC maps displayed in Fig. 5 (**c**, **d**)

The AC PET images reported here are products of reconstruction with hardware AC maps (posterior array and patient table) and the patient MRAC map determined by segmentation of fat/water Dixon MRI. Both PET and 2D-HASTE MRI images are fused in Fig. 5, using hardware TX-based AC (Fig. 5a) and CT-based AC (Fig. 5b). The CT-based AC map and TX-based AC map of the patient table are displayed with the same scale in Fig. 5 (a and b), respectively. In Fig. 6a, the RPD map produced from both CT-based and TX-based PET data shows higher RPD values towards the posterior region. The RPD between the TX-based and CT-based maps is displayed in Fig. 6b.

**Fig. 7:**
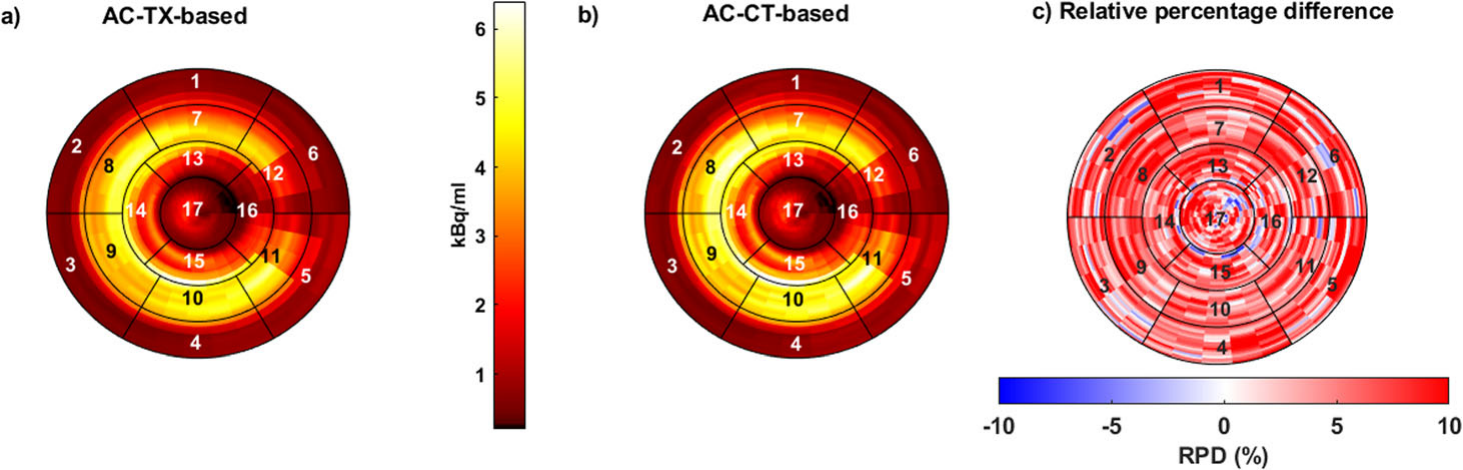
Polar plot of 17-segment according to the (AHA) model for the LV short-axis of the heart, using **a** TX-based AC, **b** CT-based AC, and **c** RPD of TX-to-CT

In Fig. 7, a polar plot of the LV PET images is shown for TX-based AC (Fig. 7a) and CT-based AC (Fig. 7b), respectively. We estimated the difference between CT-based and TX-based attenuation-corrected PET images for the following regions: apex, apical, mid, and basal which are represented by the four circles of the polar plot, from inward to outward, respectively. The RPDs for these regions were apex—3.1 ± 1.2%, apical 1.5 ± 1.1%, mid − 3.4 ± 1.0%, and basal 1.2 ± 1.1%.

## Discussion

Hardware attenuation correction based on PET transmission scans is well established and was applied using the PET/MRI scanner with few different techniques [20, 23, 26, 40]. Although the technique reported by Kawaguchi et al. was for the brain, and it was not applied to the hardware, the group suggested the need for additional techniques to incorporate MR-invisible hardware. Additionally, tissue segmentation was an issue in Kawaguchi’s work due to the three unknowns of tissue textures in the formulation.

The work done by Mollet et al. [26] utilized the time of flight (TOF) feature which allowed transmission-emission acquisition together with CT-based AC templates of patient bed and coil. Although they reported improvement in PET quantification for bone by 18.7% compare to CT-based AC, the approach was demonstrated only on a sequential TOF PET/MR system which make the approach limited to the availability of TOF feature in the system. Furthermore, the approach requires additional equipment settings (annulus phantom and pumping system for the FDG) which add complexity to patient studies and increase the radiation dose to patient. The work described in this manuscript is targeting the AC of hardware which does not require any extra scanner features, nor increases the radiation dose to the patient. The annulus method was applied by Renner et al. [32] on a head coil, where they created a coil with permanent transmission source system and reported accurate LAC in 5- to 6-min TX scan, which is similar to the time used in our study. However, in addition to the listed before, the annulus approach does not address larger hardware of the scanner itself like the patient table. Additionally, issues like consistent flow of the transmission source limits the optimum TX scan time and may increase the error in LAC estimation.

Another TX-based technique was performed by Xie et al. [40] using a fixed radioactive source (Ge-68 cylinder) where they reported improved AC of the patient table from 4.5% (CT-based AC) to 2.7% (TX-based). Their technique considered derivation of the LAC from two-point projection but they used a non-uniform TX source.

In this work, we utilized Lambert’s law, as a technique to estimate LAC at 511 keV of acrylic material with known theoretical value of LAC for validation. We simplified the technique by providing a uniform source (Ge-68 rod), the hardware profile, its voxel position, and estimated partial path length which was used in LAC estimation. The TX-based method described here was used to estimate AC maps for the posterior part of a RF phased array, and the patient table, at the regions used for simultaneous PET/MRI cardiac imaging. The produced maps from the TX-based technique were used, instead of CT-based AC, to reconstruct PET images of a volunteer offline. TX-based AC and CT-based AC for hardware have shown that PET images resulted from TX-based AC differed by less than 1% compared to the blank “truth” for all hardware cases.

The hardware used for TX-based AC was homogeneous enough, density-wise, to consider the formulation for only one unknown, which might not be the case for hardware that has larger areas with multiple densities. This technique also requires the knowledge of the hardware position and profile, and hence, it is effective for fixed hardware, but might not be as effective for mobile/flexible hardware.

The coefficient of determination of the polynomial fit (*R*^2^ = 0.9995) provides confidence in the interpolated LAC value derived from mapped energies, while the RPD between the theoretical and TX-based measured LACs validated both the acrylic cylinder attenuation coefficient and the technique for further work. The difference between NAC and AC from the blank PET image suggests that the AC procedure performed on the NAC data compensates for scatter-corrected AC PET versus non-scatter-corrected NAC PET. We also considered that the difference might represent the scatter and attenuation errors estimated by the system. Therefore, the AC data of the blank was the baseline that we used in the analysis and for comparison.

The negative RPD value between the blank and the acrylic CT-based AC images indicates overcorrection of the CT-based AC compared to the TX-based AC. This also suggests that CT-based AC of patient table is inaccurate compared to AC PET data from the blank scan. The more frequent counts observed at lower value in Fig. 4 (b, d, and f) may be interpreted as increased scattering artefacts, which may also explain the overcorrecting behaviour by CT-based AC for hardware that is previously reported in the literature [14]. Meanwhile, the RPD resulting from the comparison of in vivo PET images reconstructed by both methods suggests that CT-based AC is less accurate than the TX-based AC, which is quantified by mean RPD of 4.1 ± 0.9% and reported in Table 1.

Although this work targeted a specific region of the patient table used in cardiac imaging, the technique can be employed to produce an AC map for the entire table, or other targeted regions of the body. In our future work, we will be reporting on the development of an AC map for the full table.

## Conclusion

The TX-based AC technique described in this study resulted in similar or improved attenuation-corrected PET images compared to CT-based AC. All TX-based AC produced mean RPD of < 1%, when compared to blank data. Although the cardiac results from a patient reported here are encouraging, with changes in RPD of in vivo PET matching the improvements observed in phantom, more patients/volunteers would benefit statistical analysis. The AC maps results from the three different types of hardware demonstrated a consistent result, that is, the current CT-based AC for hardware using a bilinear model is modestly inaccurate.

Here, we have shown that the generation of TX-based AC maps using a static radioactive source and the PET/MRI system itself, for fixed hardware, is feasible. The strength of this work is that the technique allows PET and PET/MRI research sites, where access to CT or PET/CT systems is limited, to still produce highly accurate AC maps for their hardware. The TX-based hardware AC map generated using the PET/MRI system also reduces time and effort in comparison to using CT or PET/CT systems to generate hardware AC maps.

## Data Availability

The data that supports the findings of this study are available from Lawson Health Research, but restrictions apply to the availability of these data, which were used under license for the current study, and so are not publicly available. Data are however available from the authors upon reasonable request and with permission of Lawson Health Research.

## Abbreviations

AC: Attenuation correction
CT: Computed tomography
FDG: ^18^F-Fluoro-deoxy-glucose
FOV: Field of view
HASTE: Half-Fourier single-shot turbo spin-echo
LAC: Linear attenuation coefficients
LOR: Line of response
MRAC: MRI attenuation correction
MRI: Magnetic resonance imaging
NAC: Nonattenuation-corrected
PET: Positron emission tomography
QC: Quality control
RF: Radio frequency
SD: Standard deviation
SUV: Standardized uptake value
TX: Transmission
